# Efficacy and safety of intraoperative red blood cell salvage transfusion for spinal deformity surgery: a systematic review and meta-analysis

**DOI:** 10.1007/s10143-026-04520-6

**Published:** 2026-10-09

**Authors:** Kush M. Kale, Shaan Patel, Shiva A. Nischal, Pious D. Patel, Jack Jallo, James S. Harrop, Srinivas K. Prasad

**Affiliations:** 1 Present Address: Department of Physiology, Anatomy & Genetics, Medical Sciences Division, University of Oxford, Oxford, OX1 3PT UK; 2 Department of Neurological Surgery, Thomas Jefferson University Hospital, Philadelphia, PA 19107 USA

**Keywords:** Cell salvage, Blood transfusion, Blood conservation, Spinal deformity, Scoliosis, Meta-analysis

## Abstract

Spinal deformity surgery is associated with substantial blood loss and high transfusion requirements, exposing patients to the risks of allogeneic blood transfusion. Intraoperative cell salvage (ICS) is widely used in patient blood management strategies, yet its clinical efficacy and safety in spinal deformity surgery, particularly in the context of contemporary antifibrinolysis and restrictive transfusion parameters, remain uncertain. We conducted a systematic review and meta-analysis of observational studies comparing spinal deformity surgery performed with versus without ICS. PubMed, Embase, and CENTRAL were searched from inception to December 2025. Primary outcomes included transfusion volume requirements (intraoperative, postoperative, total perioperative), postoperative day 1 (POD1) hemoglobin and hematocrit, and overall complications. Secondary outcomes included the proportion of patients transfused, transfusion-related costs, and length of hospital stay. Random-effects meta-analyses were performed using restricted maximum likelihood estimation with Hartung-Knapp adjustments. Twenty-two studies comprising 2977 patients (ICS: 1851; control: 1126) were included. ICS was not associated with reductions in intraoperative (SMD 0.86, 95% CI -0.24–1.96) or total perioperative transfusion volume (SMD 1.15, 95% CI -0.75–3.05), nor with a lower likelihood of receiving any perioperative transfusion. However, ICS was associated with a modest reduction in postoperative transfusion volume (SMD 0.25, 95% CI 0.04–0.46) and higher POD1 hemoglobin (MD -0.42 g/dL, 95% CI -0.76–-0.07). No significant differences were observed in overall complications, transfusion-related costs, or length of hospital stay. In contemporary spinal deformity surgery, ICS does not meaningfully reduce overall transfusion exposure but is associated with improved early postoperative erythrocyte reserve. These findings suggest that ICS functions as a transient physiologic buffer rather than a transfusion-avoidance strategy. Its routine use is unlikely to provide substantial benefit, and future studies should explore a selective, risk-adapted role for ICS in patients with anticipated major blood loss.

**Supplementary Information:**

The online version contains supplementary material available at https://doi.org/10.1007/s10143-026-04520-6.

## Introduction

Spinal deformity encompasses a broad spectrum of structural abnormalities, including congenital malformations, adolescent idiopathic scoliosis (AIS), and adult degenerative scoliosis. AIS alone has a prevalence of 2–3% among adolescents, with approximately 0.3–0.5% progressing to severe curvatures requiring operative correction [1]. Spinal deformity in adults is even more common, affecting up to 32% of adults over 50 years, and more than two-thirds of those over 65 years [2]. Surgery remains the definitive treatment for progressive or symptomatic deformity. However, these operations are among the most physiologically demanding in spine surgery: long-segment posterior fusions, multilevel instrumentation, and complex osteotomies contribute to prolonged duration and substantial blood loss [3–5].

In this context, optimal management of intraoperative blood loss is crucial to mitigate the morbidity and mortality associated with surgical anemia and perioperative hemoglobin (Hb) decline [6]. For decades, allogeneic transfusion has remained the default approach to rapidly restore oxygen-carrying capacity, but is consistently associated with infection, transfusion reactions, and systemic complications. As a result, perioperative practice has shifted toward ‘patient blood management’ (PBM), a patient-centered approach that optimizes clinical outcomes through evidence-based blood conservation strategies [7]. PBM emphasizes preoperative anemia correction, restrictive transfusion thresholds, pharmacologic hemostasis, and autologous techniques to minimize exposure to donor blood [8].

Among autologous strategies, intraoperative cell salvage (ICS) appears conceptually attractive: shed blood aspirated from the surgical field is anticoagulated, washed, and reinfused back into the patient as a suspension of packed red blood cells (RBCs) in saline [9]. In the process, plasma proteins, platelets, and clotting factors are removed, together with activated cytokines, lipids, and debris, yielding a ‘clean’ red cell product that avoids most immunologic and infectious risks of allogeneic transfusion. Salvaged blood demonstrates high erythrocyte viability [10], relatively preserved erythrocyte morphology [11], and higher levels of 2,3-diphosphoglycerate (2,3-DPG) and ATP [12, 13] compared with stored allogeneic blood, all of which should favor microcirculatory flow and oxygen unloading. However, ICS also carries a physiological risk: infusion of large volumes of washed red cells, devoid of platelets and clotting factors, may drive dilutional coagulopathy in patients who are already bleeding excessively [14–16]. This dilemma is particularly relevant in complex spinal deformity cases, where blood loss can approach the total circulating volume [3].

The literature on ICS in spinal deformity surgery remains debatable. In pediatric scoliosis and AIS, several comparative studies have suggested that ICS can reduce exposure to allogeneic blood. Liang et al. [17] reported that ICS use halved perioperative allogeneic transfusion requirements in scoliotic patients undergoing posterior spinal fusion, with the greatest benefit in longer operations. However, robust data challenge the premise that ICS is uniformly beneficial in deformity surgery. Weiss et al. [18] found that ICS use in pediatric scoliosis did not reduce allogeneic transfusion, noting that no patient with < 500 mL blood loss received any salvaged blood. Conversely, Yang et al. [19] reported that ICS lowered allogeneic requirements in patients with relatively modest blood loss, with no demonstrable benefit in those experiencing massive hemorrhage.

Several prior reviews [20–24] have attempted to synthesize this heterogeneous evidence base. Those focused specifically on spinal deformity [21, 22] are limited by small sample size, predate contemporary PBM practices (such as routine tranexamic acid (TXA) administration), and incompletely account for concurrent autologous strategies such as preoperative autologous donation. A recent review [24] concluded that the role of ICS in spine surgery remains controversial and suggested that benefit may be greatest with high degrees of deformity, yet did not substantiate this claim with robust meta-analysis. In parallel, anesthesiology guidelines now recommend the selective use of ICS in procedures with high anticipated blood loss, framing ICS as a targeted rather than routine intervention [25].

Within this context, an updated, rigorous, and spinal deformity-specific synthesis, reflective of contemporary practice, remains warranted. We therefore conducted a systematic review and meta-analysis to evaluate the clinical efficacy and safety of ICS in spinal deformity surgery, with particular attention to perioperative hematologic parameters, transfusion requirements, and complications. By critically appraising and integrating published literature, we aim to determine whether ICS confers meaningful perioperative benefit in deformity surgery or simply adds cost and complexity without demonstrable clinical gain.

## Materials and methods

This systematic review and meta-analysis followed the Preferred Reporting Items for Systematic Reviews and Meta-Analyses (PRISMA) guidelines [26] and AMSTAR-2 methodological standards [27]. The AMSTAR-2 criteria were used to appraise the quality of previously published systematic reviews in relation to this review. The study protocol was registered a priori on the Prospective Register of Systematic Reviews (PROSPERO ID: CRD420251268337).

### Search strategy and selection criteria

A comprehensive search was performed across three databases (PubMed, Embase, and CENTRAL) from inception to 19th December 2025. The search strategy combined keywords relating to spinal deformity and ICS (Supplementary Table 1), with no language restrictions applied. Reference lists of included studies and relevant prior reviews were manually searched to ensure completeness.

Eligibility criteria included randomized or observational studies enrolling participants undergoing surgery for spinal deformity, comparing patient groups with and without ICS, and reporting at least one clinical outcome of interest. Exclusion criteria were case reports, case series, review articles, conference abstracts, technical notes, cadaveric studies, studies with no outcome data.

The predefined primary outcomes of interest were: (i) transfusion volume requirement, defined as the cumulative volume (in units) of non-salvaged RBCs transfused per patient, reported separately for the intraoperative, postoperative, and total perioperative period (intraoperative plus postoperative); (ii) postoperative day 1 (POD1) Hb and hematocrit (Hct) levels, defined as the first reported postoperative laboratory measurement obtained within 24 h of surgery, and expressed in g/dL (Hb) and percentage (%) (Hct); (iii) overall complications, analyzed as a composite outcome due to variability in individual reporting across studies, were defined as the occurrence of any postoperative adverse events reported by the primary study, including but not limited to infectious, thromboembolic, cardiopulmonary, neurologic, renal, bleeding-related, or transfusion-related complications.

Secondary outcomes were: (i) proportion of patients requiring transfusion, defined as the number of patients receiving at least one (non-salvaged) RBC transfusion during the intraoperative, postoperative, and total perioperative periods, as reported by each study; (ii) total transfusion costs, defined as the direct hospital-reported cost attributable to perioperative blood transfusion, inclusive of allogeneic blood products and, where reported, ICS-related costs; (iii) length of hospital stay (LOS).

Two authors independently performed title-and-abstract screening using Rayyan [28], followed by full text assessment. Discrepancies were resolved by consensus discussion. Inter-rater reliability was calculated using Cohen’s kappa score (κ = 0.88), indicating near-perfect agreement.

### Data analysis

Data were extracted independently by two authors. Study demographics (author, date, country, design), sample size, patient demographics, and clinical outcomes of interest stratified by ICS and control (no ICS) groups were extracted.

Two authors independently evaluated certainty of evidence for each outcome using GRADE [29] and risk of bias using Cochrane’s Risk of Bias in Non-Randomised Studies of - Interventions tool (ROBINS-I) [30].

Given high anticipated clinical and methodological heterogeneity across studies, a random effects model employing the restricted maximum-likelihood (REML) method was selected a priori for meta-analysis of pooled outcome data. Results are presented in forest plots as mean difference (MD) or standardized mean difference (SMD) for continuous outcomes, and risk ratios (RRs) for dichotomous outcomes. Because transfusion volume is a discrete, ratio-scale variable, volume outcomes were additionally pooled as the ratio of means (RoM), with the same random-effects model applied on the log scale. Corresponding 95% confidence intervals (CIs) were derived using the Hartung-Knapp-Sidik-Jonkman (HKSJ) method [31] for each outcome. Heterogeneity was assessed with Cochran’s Q test (*P* < 0.05 considered statistically significant) and I^2^ statistics (> 25% considered significant). Under this random-effects model, individual study weights were derived from the inverse of the sum of the within-study and between-study variance; study weight therefore reflects the precision of each estimate rather than sample size alone, and weights converge toward equality as between-study heterogeneity increases.

Leave-one-out sensitivity analyses were conducted to investigate whether any particular study had a disproportionate contribution to statistically significant results. Publication bias was assessed using funnel plots for outcomes with ≥ 10 studies, in line with Cochrane guidance [32]. All analyses were performed using Review Manager (RevMan) Version 9.16.1 [33].

Trial sequential analysis was performed for primary outcomes to evaluate the robustness of the cumulative evidence and to control for random error; required information sizes (RIS) were estimated using a D^2^-based heterogeneity adjustment (two-sided α = 0.05; 80% power).

## Results

**Fig. 1:**
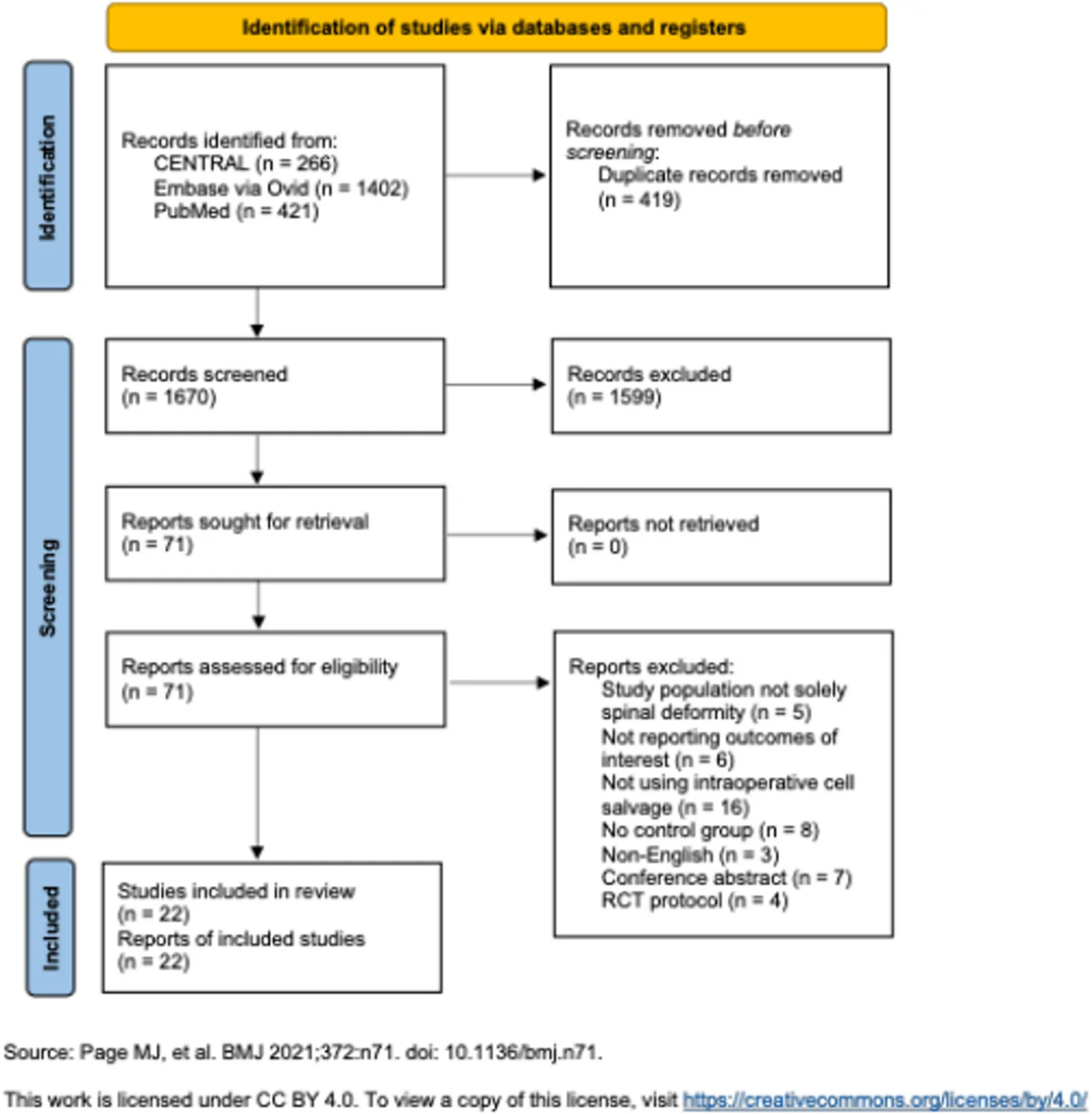
PRISMA flow diagram of study selection

A total of 1670 records were identified, of which 22 studies met inclusion criteria (Fig. 1). These comprised 18 retrospective [18, 34–50] and 4 prospective cohort studies [17, 51–53], collectively including 2977 patients (ICS: 1851; control: 1126) (Table 1). Study sample sizes ranged from 33 to 580 participants. Reported mean ages ranged from 12.8 to 59.2 years in control (non-ICS) cohorts and from 12.8 to 56.8 years in ICS cohorts. The proportion of female patients ranged from 25.5% to 90.0% in control groups and from 23.2% to 92.7% in ICS cohorts. Estimated blood loss ranged from 293.0 to 4800.0 mL in control cohorts, and 474.0 to 4100.0 mL in ICS cohorts.

**Table 1:** Characteristics of included studies

Author (Year)	Country	Study design	Sample size		Age, in years (mean ± SD or mean (range))		Sex (% female)	
			No ICS	ICS	No ICS	ICS	No ICS	ICS
Akgul (2014)	Turkey	RCS	17	16	18.7 ± 6.8	17.1 ± 3.9	84.8%	
Al-Mohrej (2020)	Saudi Arabia	RCS	71	71	NR	NR	NR	NR
Behrman (1992)	USA	PCS	25	25	23.2	22	88.0%	84.0%
Bowen (2010)	USA	RCS	21	33	14.8 ± 1.9	14.3 ± 1.5	NR	NR
Canan (2012)	USA	RCS	30	150	NR	NR	66.7%	60.7%
Chi (2023)	China	RCS	203	199	12.8 ± 1.8	12.8 ± 2.3	66.5%	64.8%
Choi (2019)	Korea	RCS	32	81	55.6 ± 24.2	53.5 ± 24.7	78.1%	77.8%
Ersen (2012)	Turkey	RCS	22	23	13.8 ± 2.0	14.7 ± 1.5	77.3%	87.0%
Gum (2017)	USA	RCS	153	427	59.2 ± 14.8	56.8 ± 14.7	25.5%	23.2%
Hansen (1987)	Germany	RCS	17	35	NR	NR	NR	NR
Katranitsa (2018)	Greece	RCS	25	61	15.8 ± 2.2	16.5 ± 2.4	64.0%	80.3%
Kurland (2025)	USA	PCS	73	101	48.1 ± 25.4	52.1 ± 25.3	65.8%	52.5%
Lennon (1987)	USA	RCS	28	142	28.4 ± 14.4	25.1 ± 14.2	NR	NR
Liang (2015)	China	PCS	55	55	16.8 ± 7.0	15.5 ± 5.6	81.8%	72.7%
Loughenbury (2016)	UK	RCS	37	30	15.0 (8.0-33.0)	16.0 (9.0-57.0)	NR	NR
Miao (2014)	China	RCS	60	60	14.9 ± 2.4	14.7 ± 2.2	61.7%	63.3%
Mirza (2009)	UK	RCS	33	104	16.4	16.4	69.7%	82.7%
Oliveira (2017)	Brazil	RCS	15	27	17.4 ± 4.9	18.7 ± 5.3	86.7%	92.6%
Siller (1996)	USA	RCS	55	55	14.1 (10.7-16.8)	14.3 (11.0-16.9)	90.0%	92.7%
Wahl (2021)	USA	RCS	100	69	14.6 ± 1.9		78.1%	
Weiss (2007)	USA	RCS	37	58	14.4 ± 2.1	14.0 ± 2.2	67.6%	77.6%
Zhou (2014)	China	PCS	17	29	19.5 ± 5.9	20.4 ± 5.4	NR	NR

Author (Year)	Number of levels fused		Preoperative Cobb angle (°)		Preoperative hemoglobin (g/dL)		Preoperative hematocrit (%)		Estimated blood loss (mL)	
	No ICS	ICS	No ICS	ICS	No ICS	ICS	No ICS	ICS	No ICS	ICS
Akgul (2014)	12.8 ± 1.5	12.9 ± 1.5	NR	NR	13.1 ± 1.6	12.2 ± 1.5	NR	NR	NR	NR
Al-Mohrej (2020)	NR	NR	NR	NR	NR	NR	NR	NR	NR	NR
Behrman (1992)	NR	NR	NR	NR	NR	NR	37.6	38.6	942.0	1005.0
Bowen (2010)	9.8 ± 1.8	9.8 ± 1.7	64.0 ± 19.0	62.0 ± 11.0	13.2 ± 1.3	13.3 ± 0.9	NR	NR	NR	NR
Canan (2012)	NR	NR	NR	NR	NR	NR	NR	NR	470.8 (75.0-3000.0)	473.6 (50.0-3000.0)
Chi (2023)	8.7 ± 2.8	9.2 ± 2.9	59.4 ± 20.7	61.0 ± 18.9	13.4 ± 1.2	13.5 ± 1.1	39.4 ± 3.4	39.7 ± 2.2	1082.6 ± 412.3	1183.7 ± 427.5
Choi (2019)	8.3 ± 2.2	9.6 ± 2.8	NR	NR	13.2 ± 1.7	12.9 ± 1.8	NR	NR	1098.4 ± 617.5	953.7 ± 774.5
Ersen (2012)	11.5 ± 1.4	12.0 ± 1.1	54.7 ± 12.8	55.3 ± 11.5	13.6 ± 1.2	13.5 ± 0.9	40.1 ± 3.6	39.8 ± 2.5	1006.0 ± 278.0	973.0 ± 245.0
Gum (2017)	11.4 ± 3.7	11.9 ± 3.9	NR	NR	NR	NR	NR	NR	986.8 ± 1198.1	1992.0 ± 1557.1
Hansen (1987)	NR	NR	NR	NR	NR	NR	NR	NR	4800.0 ± 1600.0	4100.0 ± 1400.0
Katranitsa (2018)	10.2 ± 3.0	9.6 ± 1.9	NR	NR	13.1 ± 1.7	11.6 ± 1.1	39.8	35.3	NR	NR
Kurland (2025)	8.3 ± 3.5	11.2 ± 3.7	NR	NR	NR	NR	NR	NR	1247.6 ± 1553.5	2111.4 ± 1638.9
Lennon (1987)	7.4 ± 2.2	7.7 ± 2.1	NR	NR	13.5 ± 1.4	13.5 ± 1.3	NR	NR	NR	NR
Liang (2015)	10.9 ± 3.0	11.3 ± 2.7	NR	NR	13.7 ± 1.0	13.7 ± 1.0	40.5 ± 2.8	40.7 ± 2.7	696.8 ± 374.3	767.4 ± 362.3
Loughenbury (2016)	NR	NR	NR	NR	13.1 ± 0.5	13.5 ± 0.6	NR	NR	NR	NR
Miao (2014)	9.9 ± 3.9	9.9 ± 4.2	94.7 ± 25.6	90.4 ± 30.0	12.9 ± 1.3	13.0 ± 1.5	39.8 ± 4.6	40.4 ± 8.8	2135.8 ± 1303.9	2185.8 ± 1315.4
Mirza (2009)	5.1	5.1	NR	NR	13.4	13.5	39.0	41.0	NR	NR
Oliveira (2017)	NR	NR	NR	NR	13.1 ± 1.3	13.3 ± 1.1	39.3 ± 3.5	39.3 ± 2.8	NR	NR
Siller (1996)	11.1 (7.0-14.0)	11.5 (7.0-14.0)	NR	NR	NR	NR	35.9 (29.9-45.6)	35.3 (28.2-46.1)	1084.0 (242.0-2477.0)	823.0 (186.0-1587.0)
Wahl (2021)	11.1 ± 2.4	12.1 ± 1.4	54.7	58.5	NR	NR	NR	NR	293.0 (85.0-700.0)	528.0 (200.0-1800.0)
Weiss (2007)	12.7 ± 2.9	11.2 ± 3.3	63.1 ± 11.4	63.9 ± 17.6	13.4 ± 0.9	13.5 ± 1.2	39.8 ± 2.7	39.4 ± 4.8	656.0 ± 654.1	781.2 ± 727.8
Zhou (2014)	10.2 ± 1.2	10.6 ± 1.3	69.2 ± 16.8	68.2 ± 15.8	11.5 ± 1.1	12.1 ± 1.4	32.0 ± 5.0	31.0 ± 4.0	835.3 ± 167.5	812.1 ± 152.7

Summary of demographic and operative characteristics for all comparative studies included in the meta-analysis. Columns report study author and publication year, country of study origin, study design, total sample size per surgical group, age distribution, sex distribution, number of vertebral levels fused, preoperative Cobb angle, preoperative hemoglobin and hematocrit, and estimated blood loss. Abbreviations: *ICS *intraoperative cell salvage, *RCS *retrospective cohort study, *PCS *prospective cohort study, *NR *not reported

Methodological appraisal using AMSTAR-2 rated the present review as high quality (Supplementary Table 2).

Preferred Reporting Items for Systematic Reviews and Meta-Analyses (PRISMA) flow diagram outlining the study selection process. The number of records identified, screened, assessed for eligibility, and included in the final analysis are detailed at each stage.

### Transfusion volume requirements

**Fig. 2:**
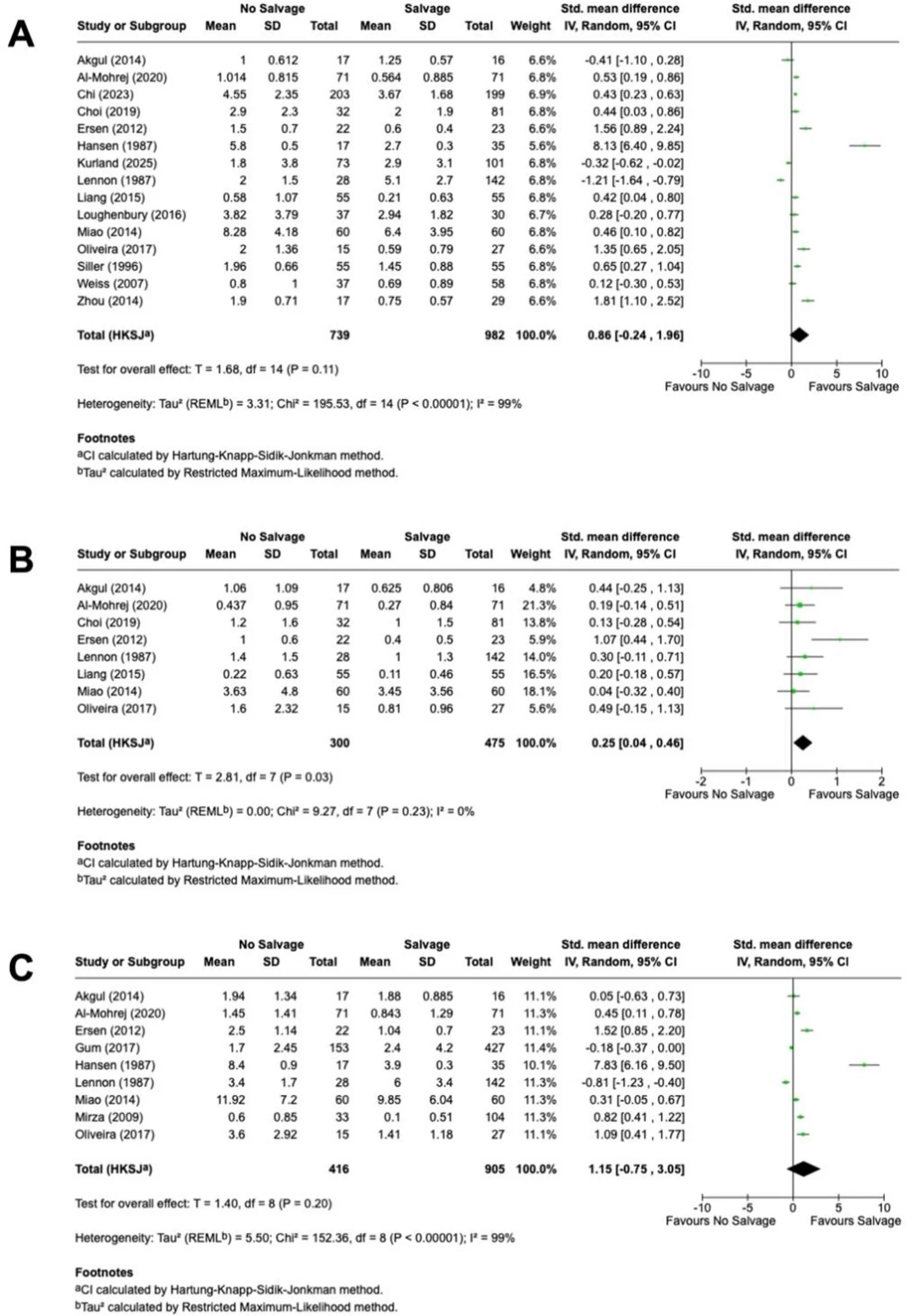
Transfusion volume requirements with versus without ICS use. Forest plot comparing the standardized mean difference (random effects model) in transfusion volume requirements during the (**A**) intraoperative, (**B**) postoperative, and (**C**) total perioperative periods in patients receiving (‘Salvage’) or not receiving (‘No Salvage’) intraoperative cell salvage. Individual study estimates are shown as squares proportional to study weight. Horizontal lines indicate 95% confidence interval. Diamond represents pooled estimate for overall effect. Negative values indicate higher transfusion requirements in the salvage group. IV = inverse variance.

Intraoperative transfusion requirement was reported in 15 studies (68.2%) [17, 18, 34, 35, 38–40, 42, 44–46, 48, 49, 52, 53] with a pooled SMD of 0.86 (95% CI: -0.24–1.96; *P* = 0.11; I² = 99.0%) (Fig. 2A). Postoperative transfusion volume was reported in 8 studies (36.4%) [17, 34, 35, 39, 40, 44, 46, 48]. ICS use was associated with a significant reduction in postoperative transfusion volume (SMD 0.25, 95% CI: 0.04–0.46; *P* < 0.05; I² = 0.0%) (Fig. 2B). Sensitivity analysis confirmed stability of the effect estimate. Total perioperative transfusion volume was reported in 9 studies (40.9%) [34, 35, 40–42, 44, 46–48], with a pooled SMD of 1.15 (95% CI: -0.75–3.05; *P* = 0.20; I² = 99.0%) (Fig. 2C). Re-expressing transfusion volume outcomes as the RoM yielded results concordant with SMD analyses (Supplementary Table 3). Confidence intervals were substantially more stable and residual heterogeneity markedly lower on the ratio scale, indicating that much of the extreme heterogeneity observed under SMD reflects between-study differences in measurement scale rather than true variation in treatment effect.

### Postoperative hematological parameters

**Fig. 3:**
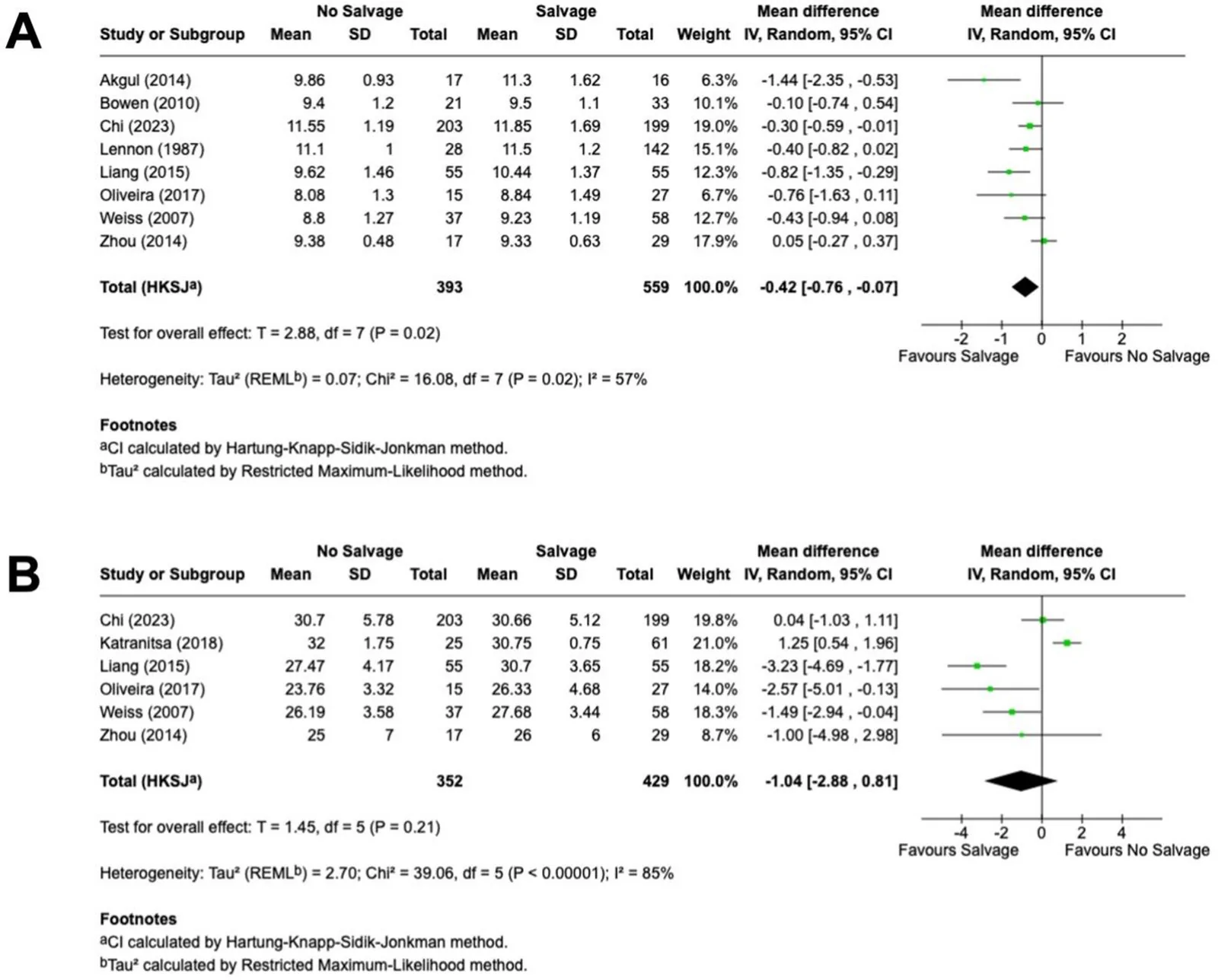
Postoperative hematologic parameters with versus without ICS use. Forest plot comparing the mean difference (random effects model) in (**A**) hemoglobin and (**B**) hematocrit values on postoperative day 1 (POD1) in patients receiving (‘Salvage’) or not receiving (‘No Salvage’) intraoperative cell salvage. Individual study estimates are shown as squares proportional to study weight. Horizontal lines indicate 95% confidence interval. Diamond represents pooled estimate for overall effect. Negative values indicate higher hematologic parameters in the salvage group. IV = inverse variance.

POD1 Hb levels were reported in 8 studies (36.4%) [17, 18, 34, 36, 38, 44, 48, 53]. ICS was associated with significantly higher POD1 Hb levels (MD -0.42 g/dL, 95% CI: -0.76–-0.07 g/dL; *P* < 0.05; I² = 57.0%) (Fig. 3A). Sensitivity analysis confirmed stability of the effect estimate. POD1 Hct was reported in 6 studies (27.3%) [17, 18, 38, 43, 48, 53], with a pooled MD of -1.04% (95% CI: -2.88–0.81%; *P* = 0.21; I² = 85.0%) (Fig. 3B).

### Overall complications

**Fig. 4:**
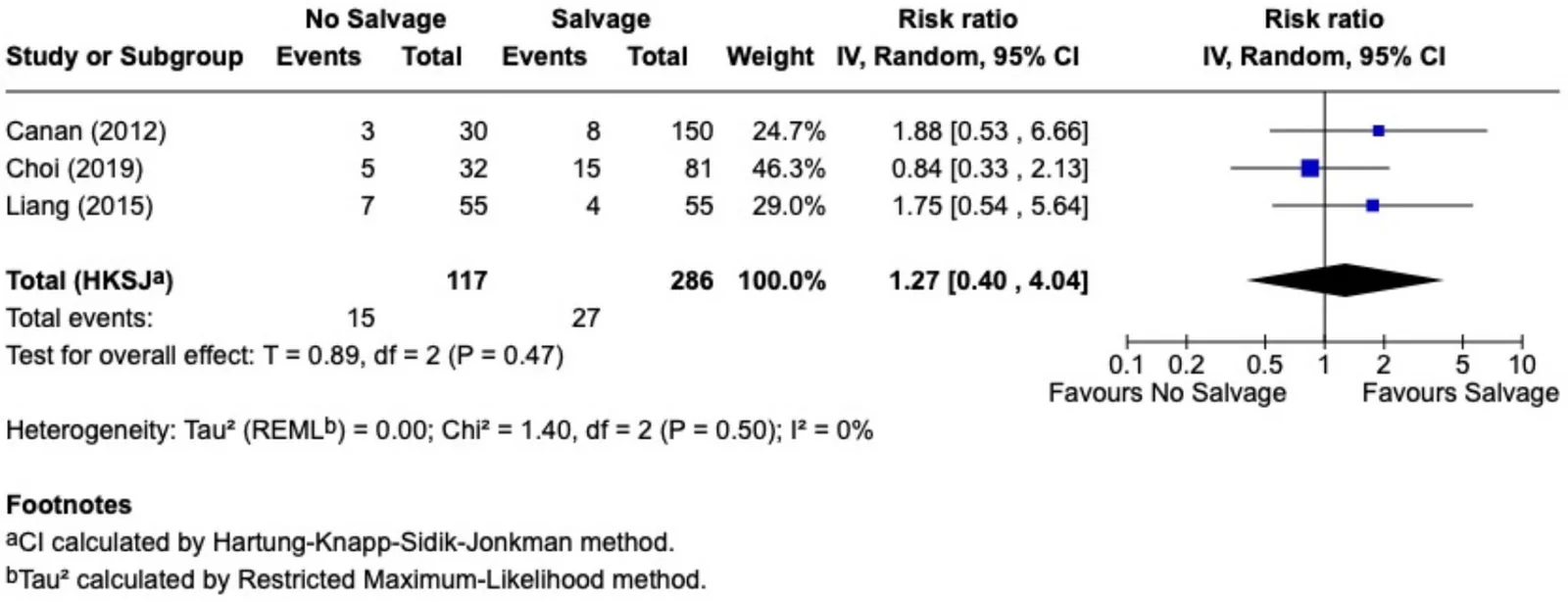
Overall complications with versus without ICS use. Forest plot comparing the risk ratio (random effects model) of overall complications following surgery in patients receiving (‘Salvage’) or not receiving (‘No Salvage’) intraoperative cell salvage. Individual study estimates are shown as squares proportional to study weight. Horizontal lines indicate 95% confidence interval. Diamond represents pooled estimate for overall effect. IV = inverse variance.

Overall complications were reported in 3 studies (13.6%) [17, 37, 39]. Complications occurred in 12.8% of control patients (15/117) and 9.4% of ICS patients (27/286). The pooled RR was 1.27 (95% CI: 0.40–4.04; *P* = 0.47; I² = 0.0%) (Fig. 4).

### Proportion of patients requiring transfusion

The proportion of patients receiving intraoperative transfusion was reported in 6 studies (27.3%) [35, 36, 43, 45, 48, 50]. Transfusion was administered in 39.4% of control patients (106/269) and 34.1% of ICS patients (87/255). The pooled RR was 1.25 (95% CI: 0.38–4.13; *P* = 0.65; I² = 94.0%) (Supplementary Fig. 1A). The proportion of patients receiving postoperative transfusion was reported in 4 studies (18.2%) [35, 47, 48, 50]. Transfusion was administered in 20.5% of control patients (45/219) and 12.2% of ICS patients (33/271). The pooled RR was 1.95 (95% CI: 0.53–7.20; *P* = 0.20; I² = 73.0%) (Supplementary Fig. 1B). The proportion of patients receiving perioperative transfusion was reported in 5 studies (22.7%) [17, 35, 36, 41, 46]. Transfusion was administered in 56.7% of control patients (204/360) and 55.1% of ICS patients (356/646). The pooled RR was 1.43 (95% CI: 0.67–3.06; *P* = 0.27; I² = 95.0%) (Supplementary Fig. 1C).

### Healthcare resource utilization

Transfusion cost was reported in 3 studies (13.6%) [38, 46, 48]. The pooled MD was -$47.30 USD (95% CI: -572.66–478.06 USD; *P* = 0.74; I² = 99.0%) (Supplementary Fig. 2). LOS was reported in 4 studies (18.2%) [43, 45, 46, 48]. The pooled MD was − 0.06 days (95% CI: -0.77–0.65 days; *P* = 0.81; I² = 0.0%) (Supplementary Fig. 3).

### Trial sequential analysis

TSA indicated firm evidence for postoperative transfusion volume and POD1 Hb, with both crossing the conventional boundary and RIS. Conversely, intraoperative and total perioperative transfusion volume, POD1 Hct, and overall complications remained inconclusive (Supplementary Fig. 4).

### Publication bias, risk of bias, and quality assessment

Funnel plot inspection revealed no marked asymmetry, although 6 of 15 studies lay outside the 95% CI limits (Supplementary Fig. 5), likely attributable to substantial inter-study heterogeneity. Egger’s regression test demonstrated no evidence of small-study effects (*P* = 0.082).

Among the 22 included studies, 1 study was judged to be low risk of bias, 2 studies at moderate risk, and 19 studies at serious risk, with confounding representing the most frequent methodological concern, followed by bias due to missing data (Supplementary Table 4). Certainty of evidence, assessed using the GRADE framework, was judged to be moderate across primary outcomes, with downgrading primarily due to risk of bias (Supplementary Table 5).

Quality appraisal of 3 previous systematic reviews relevant to this topic identified all as critically low quality according to AMSTAR-2 criteria (Supplementary Table 6).

## Discussion

This systematic review and meta-analysis, synthesizing 22 spinal deformity cohorts, represents the most contemporary and statistically robust evaluation of ICS to date. Across 2977 patients, ICS did not significantly reduce intraoperative or overall perioperative transfusion requirements, nor the proportion of patients transfused, but did yield a modest reduction in postoperative transfusion requirement and higher POD1 Hb. These pooled estimates should nonetheless be interpreted with caution: most included studies were retrospective and at serious risk of bias, with confounding by indication (i.e., preferential use of ICS in higher blood loss cases) the predominant methodological concern.

Our findings are directionally consistent with prior meta-analyses. In a scoliosis-focused synthesis, Liu et al. [21] reported ICS significantly reduced perioperative and postoperative allogeneic transfusion volumes and increased POD1 Hb and Hct, while leaving intraoperative transfusion volume largely unchanged. Cheriyan et al.’s [23] broader analysis (including all types of spine surgery) found that ICS did not reduce the incidence of perioperative transfusion, but modestly decreased intraoperative transfusion volume by ~ 0.8 units. Our results extend these observations by focusing on spinal deformity (where blood loss and transfusion risk are among the highest in spine surgery), incorporating more recent cohorts, and demonstrating a consistent signal of improved early postoperative erythrocyte reserve (despite no decrease in overall transfusion exposure). A pattern emerges of ICS functioning less as a transfusion-avoidance strategy and more as a perioperative erythrocyte buffer.

### Intraoperative blood management: competing physiologic trade-offs

Despite being a cornerstone of perioperative care, allogeneic transfusion remains constrained by adverse effect profiles. During storage, donor RBCs accumulate a set of biochemical and biomechanical changes, collectively termed the ‘storage lesion’, including ATP and 2,3-DPG depletion, oxidative stress, and membrane damage that often precipitates hemolysis [54, 55]. Consequently, a significant fraction of these lesioned RBCs are rapidly cleared by the reticuloendothelial system, with release of cell-free Hb and non-transferrin bound iron -moieties that scavenge nitric oxide, promote endothelial dysfunction, and drive oxidative stress in vivo, disrupting microvascular oxygen delivery to already hypoxic tissues. In parallel, however, a growing body of evidence has uncovered the immunomodulatory consequences of allogeneic transfusion, which are linked to increased susceptibility to infection, organ dysfunction, and tumor progression [56].

ICS is conceptually attractive as it bypasses the storage lesion entirely and avoids donor alloantigen exposure. Salvaged blood is autologous, normothermic, and typically reinfused within hours of collection. Levels of 2,3-DPG and ATP are preserved [12, 13], membrane morphology remains more physiological [11], and the rheological properties of salvaged cells are superior to those of allogeneic cells [57].

However, ICS use introduces its own set of limitations. By design, ICS systems collect, concentrate, and wash shed blood to produce a suspension of packed RBCs, devoid of platelets, coagulation factors, and plasma proteins [9]. This process is inherently traumatic to erythrocytes such that RBCs sustain greater sublethal and lethal injury, which increases mechanical fragility [58], results in greater free Hb concentrations compared to circulating or allogeneic blood [59], and produces a measurable degree of hemolysis even under optimal conditions [60]. Moreover, in patients with major blood loss, infusion of salvaged blood represents a hemostatic challenge that may exacerbate dilutional coagulopathy unless accompanied by timely fibrinogen and platelet replacement [14–16]. In the context of spinal deformity, where there is a greater incidence of baseline coagulative derangement [61, 62], even modest dilutional effects can be clinically meaningful. Consistent with this view, a recent spinal deformity trial reported a significantly higher incidence of postoperative thrombocytopenia in patients receiving ICS that persisted after adjusting for covariates [52].

Collectively, these data suggest ICS mitigates the chronic storage lesion of banked blood in exchange for an acute ‘trauma lesion’ induced by suction, centrifugation, and washing, which may be equally deleterious and underlie shorter salvaged RBC lifespans [63]. Thus, in spinal deformity surgery, ICS appears to modestly augment early postoperative erythrocyte reserve (reflected by higher POD1 Hb) without materially altering overall transfusion exposure, though this benefit may be transient if RBC survival is curtailed. Whether this short-term hematologic advantage translates into clinically meaningful differences remains unclear due to sparse and inconsistent reporting. Nonetheless, when considering the known prognostic value of postoperative Hb in spine surgery [64, 65], the ability of ICS to attenuate early postoperative anemia may confer a cumulative benefit that is not fully captured by standard transfusion-centric endpoints.

### Diminishing returns in the era of antifibrinolysis

ICS did not reduce intraoperative or total perioperative transfusion volume, nor the proportion of patients transfused. An important consideration is the widespread adoption of multimodal blood-sparing strategies in contemporary spinal deformity surgery (most notably TXA) within a broader PBM framework. In this setting, ICS no longer functions in isolation, but as one component of an integrated hemostatic strategy.

Many foundational studies evaluating ICS in spine surgery predate routine TXA use and contemporary PBM protocols. At the time, transfusion thresholds were more liberal and baseline blood loss during long-segment fusions was substantially higher [42]. In contrast, current practice is characterized by near-ubiquitous TXA administration and protocolized blood conservation [66]. Multiple systematic reviews and meta-analyses demonstrate that TXA reduces intraoperative blood loss by approximately 30–40% and decreases perioperative allogeneic transfusion without increasing thrombotic risk [67–69]. Cost-effectiveness analyses further show that TXA is economically favorable, largely through its impact on transfusion avoidance [70]. Against this backdrop, the incremental contribution of ICS is inherently limited. As baseline blood loss is lowered by PBM measures, the absolute volume available for salvage decreases, restricting the capacity of ICS to meaningfully displace allogeneic transfusion. This pattern is consistent with prior meta-analysis [23], which show modest reductions in transfused units (typically less than one unit per patient) without significant effects on the proportion of patients transfused.

Current guidelines reflect this principle of ‘selective yield’. The Association of Anaesthetists [25] recommends cell salvage for procedures with expected blood loss exceeding 500 mL or 10% of circulating volume, explicitly positioning ICS as a targeted rather than routine intervention. Our findings are consistent with this position: in the presence of TXA and established PBM protocols, routine use of ICS in low or moderate blood loss deformity cases is unlikely to confer substantial additional benefit. We did not, however, directly compare pre- and post-PBM cohorts, and this interpretation therefore reflects a hypothesis generated by the temporal pattern of the evidence rather than a finding of the present analysis.

### Cost, value, and patient selection

In pooled analysis, ICS was not associated with a significant reduction in transfusion-related costs. Nonetheless, interpretation is limited by sparse reporting and substantial heterogeneity in costing methods, institutional pricing, and accounting of fixed versus variable costs. As such, the apparent pooled cost signal neutrality should be interpreted as reflecting context-dependent value.

Several focused economic evaluations suggest that the cost-effectiveness of ICS is highly sensitive to case-mix and anticipated blood loss. In a prospective multicenter cohort of adult spinal deformity surgery, Gum et al. [41] demonstrated that ICS became cost-efficient once estimated blood loss exceeded approximately 600 mL, despite no reduction in the proportion of patients receiving transfusion. In pediatric scoliosis, Weiss et al. [18] estimated that the threshold for cost neutrality was reached when expected blood loss exceeded 500 mL, whereas Chi et al. [38] reported a threshold of 1500 mL. Although exact thresholds vary, the unifying observation is that economic value is driven primarily by blood loss severity. Practically, these data argue against indiscriminate use of ICS in deformity surgery, particularly in lower-risk constructs or in centers where PBM pathways are already well-established [71]; in such settings, the marginal benefit of ICS is unlikely to offset the fixed and operational costs associated with its use.

Conversely, ICS may offer both clinical and economical advantage when selectively applied to patients at high risk of major hemorrhage, including those undergoing long-segment constructs, three-column osteotomies, revision deformity surgery, or procedures in which allogeneic transfusion is undesirable or contraindicated [72]. Here, ICS functions less as a cost-saving universal strategy and more as a risk-mitigating adjunct within a targeted PBM framework. We were, however, unable to test these high-risk subgroups directly: the included studies did not report outcomes stratified by construct length, osteotomy, revision status, or anticipated blood loss, so the greater benefit proposed for these patients remains a hypothesis to be confirmed in adequately powered, prospectively stratified studies.

### Study limitations and sources of heterogeneity

Our findings should be interpreted in light of several important limitations, most of which reflect the inherent complexity of studying blood management interventions in spinal surgery. First, most included studies were retrospective and observational, introducing substantial potential for confounding by indication. In practice, ICS is rarely deployed at random; rather, it is preferentially used in cases where greater blood loss is anticipated. This pattern was explicitly documented in several included cohorts [18, 46], and aligns with predictive models which consistently identify longer fusion length, osteotomy use, and lower preoperative Hb as strong determinants of transfusion risk [73–75]. Consequently, patients receiving ICS often represent a systematically higher risk subgroup, which may attenuate or obscure observable treatment effects. Second, there was considerable inconsistency in how ICS and control groups were defined across studies. Some series employed an ‘intention-to-treat’ approach, classifying patients as ICS if a cell salvage system was available during surgery, irrespective of whether salvaged blood was ultimately reinfused. Others defined exposure based solely on receipt of salvaged blood. Given incomplete and heterogeneous reporting, we retained original study definitions rather than imposing post-hoc reclassification, acknowledging that this variability likely contributed to inter-study heterogeneity. Third, co-interventions related to perioperative blood management varied widely across studies. These included preoperative autologous blood donation [43, 49, 53], hypotensive anesthesia [44, 53], and TXA administration [45, 48, 50], each of which independently influences blood loss and transfusion requirements. Finally, outcome definitions and reporting windows were non-uniform. The ‘postoperative’ window was variably defined, ranging from the first 24 h to hospital discharge, and the term ‘perioperative’ was inconsistently applied. In addition, the volume of a ‘unit’ of blood varies across institutions [76]. To address these issues and improve comparability, we analyzed continuous outcomes using standardized mean differences where appropriate, though this approach necessarily trades interpretability for statistical consistency. We further note that transfused RBC volume is a discrete, typically right-skewed count variable, whereas the SMD assumes approximately normally distributed data. Since included studies reported only patient-level means and standard deviations rather than event counts with person-time, an incidence rate ratio could not be applied. We therefore re-expressed volume outcomes as the RoM, which yielded results consistent with the primary analysis. Included studies also spanned publication years from 1987 to 2025 and with a wide age range, encompassing markedly different transfusion eras and patient populations that differ in circulating blood volume, deformity morphology, and transfusion thresholds. Several cohorts predate routine TXA and contemporary PBM, and inconsistent reporting of these co-interventions and of patient age precluded formal stratification by PBM era or by pediatric versus adult status; future prospective studies should differentiate these groups.

Reflecting these methodological and clinical sources of variability, several primary outcomes demonstrated very high statistical heterogeneity, suggesting that observed between-study differences reflect true underlying variation rather than sampling error alone [77]. To mitigate this, we employed a random-effects modeling approach using the REML estimator and HKSJ adjustment, widely regarded as more statistically robust than traditional DerSimonian and Laird methods, particularly in the presence of high heterogeneity and a limited number of studies [78]. Clinically, heterogeneity of this magnitude means the pooled point estimates for these outcomes cannot be read as a single effect applicable to every patient; the true effect of ICS is likely to vary with case complexity, blood-loss burden, and local transfusion practice, favoring an individualized rather than uniform approach. The evidence base also remains underpowered for several outcomes, particularly overall complications, reported in only 3 of 22 studies. Relatedly, postoperative transfusion volume showed a significant reduction with ICS while total perioperative volume did not, despite the latter nominally including the former. Importantly, these outcomes drew on non-identical and only partially overlapping sets of studies. The present non-significant results should therefore be interpreted as inconclusive rather than as evidence of no effect. We regard a reduction of approximately one allogeneic RBC unit per patient, or a 10% absolute reduction in the proportion of patients transfused, as a clinically meaningful benefit against which these estimates should be judged; the confidence intervals for the non-significant outcomes remain compatible with effects of this magnitude.

### Clinical implications and future directions

From a clinical perspective, our findings are consistent with a selective and context-dependent role for ICS in spinal deformity surgery. ICS should not be viewed as a strategy to eliminate allogeneic transfusion requirements in most patients. Rather, it appears to function as a physiologic buffer that modestly augments early postoperative Hb and reduces postoperative allogeneic transfusion volume, at the expense of potential dilutional coagulopathy and mechanical erythrocyte injury. These competing effects underscore that ICS is neither uniformly beneficial nor redundant but instead exerts a limited and situational impact within modern perioperative care. In practical terms, three implications follow. First, ICS is best reserved for deformity procedures with a high anticipated risk of major blood loss. Second, decisions regarding ICS deployment should be embedded within a comprehensive PBM framework, rather than considered in isolation. Third, evaluation of blood management strategies should extend beyond transfusion volume alone to include postoperative anemia severity, recovery trajectories, and downstream complications (including patient-reported), which may better capture clinically meaningful effects than binary transfusion endpoints.

Future research should aim to refine this selective paradigm. Priorities include prospective, deformity-specific trials comparing PBM approaches with and without ICS in clearly-defined high blood loss subgroups, rather than unselected surgical populations. Mechanistic studies are needed to determine whether laboratory evidence of RBC injury during cell salvage translates into clinically measurable hemolysis and reduced erythrocyte survival after reinfusion. Finally, robust cost-effectiveness analyses incorporating modern PBM practices, will be essential to establish rational thresholds for ICS deployment. Together, such work will determine whether ICS meaningfully improves outcomes in the subset of deformity patients most vulnerable to transfusion-related morbidity, or whether its principal role in contemporary practice remains limited to modest modulation of early postoperative Hb within an increasingly PBM-dominated perioperative landscape.

## Conclusion

In summary, ICS was not associated with reductions in intraoperative or total perioperative transfusion exposure, nor with a lower likelihood of receiving allogeneic blood. Instead, its principal measurable effect was a modest improvement in early postoperative erythrocyte reserve, reflected by higher POD1 Hb and reduced postoperative transfusion volume. These findings suggest that in contemporary deformity practice, ICS functions less as a transfusion-avoidance strategy and more as a transient physiologic buffer within the perioperative period.

These findings generate the hypothesis that ICS is most useful in patients at high risk of major hemorrhage, where it may provide incremental physiological reserve or mitigate transfusion exposure, particularly when alternative strategies are insufficient or contraindicated. Future prospective, deformity-specific studies integrating mechanistic, clinical, and economic endpoints will be essential to refine patient selection and determine whether ICS confers clinically meaningful benefit beyond modest modulation of early postoperative Hb in an increasingly PBM-dominated perioperative landscape.

## Supplementary Information

Below is the link to the electronic supplementary material.

Supplementary Material 1

Supplementary Material 2

## Data Availability

No datasets were generated or analysed during the current study.

## Abbreviations

2,3-DPG: 2,3-diphosphoglycerate
ATP: Adenosine triphosphate
AIS: Adolescent Idiopathic Scoliosis
CI: Confidence Interval
Hct: Hematocrit
Hb: Hemoglobin
ICS: Intraoperative Cell Salvage
MD: Mean Difference
PBM: Patient Blood Management
POD1: Postoperative Day 1
RBC: Red Blood Cell
ROBINS-I: Risk of Bias in Non-Randomised Studies of - Interventions tool
RR: Risk Ratio
SMD: Standardized Mean Difference
TXA: Tranexamic Acid
