# Supplementary material for: Efficacy and safety of intraoperative red blood cell salvage transfusion for spinal deformity surgery: a systematic review and meta-analysis
Source: Neurosurg Rev. 2026 Oct 9;49(1):604. doi: 10.1007/s10143-026-04520-6 (PMC13649948; doi:10.1007/s10143-026-04520-6)
Supplement: Supplementary file 2 [file 10143_2026_4520_MOESM2_ESM.docx]

**Supplementary Digital Content**

Title: Efficacy and safety of intraoperative red blood cell salvage transfusion for spinal deformity surgery: a systematic review and meta-analysis

Authors:

Kush M. Kale^1^*, Shaan Patel^2^*, Shiva A. Nischal^1^, Pious D. Patel^2^, Jack Jallo^2^, James S Harrop^2^, Srinivas K. Prasad^2^

1. Department of Physiology, Anatomy & Genetics, Medical Sciences Division, University of Oxford, OX1 3PT, UK
2. Department of Neurological Surgery, Thomas Jefferson University Hospital, Philadelphia, PA 19107, USA.

*Co-First Authors: KMK & SP

Corresponding Author:

Name: Kush M. Kale

Current Institution: Department of Physiology, Anatomy & Genetics, Medical Sciences Division, University of Oxford, Oxford, UK

Email: [kush.kale@sjc.ox.ac.uk](mailto:kush.kale@sjc.ox.ac.uk)

| **Database** | **Search Strategy** |
| --- | --- |
| PubMed  (n=421) | ("Spinal Fusion"[MeSH] OR "Scoliosis"[MeSH] OR "Kyphosis"[MeSH] OR "Spinal Curvatures"[MeSH] OR "Spine/surgery"[MeSH] OR "spinal deformity" OR "adult spinal deformity" OR "spine surgery" OR scoliosis OR kyphosis OR lordosis OR "interbody fusion" OR "instrumented fusion")    AND  ("Erythrocyte Transfusion"[MeSH] OR "Blood Transfusion, Autologous"[MeSH] OR “Operative Blood Salvage”[MeSH] OR "autologous blood transfusion" OR "cell salvage" OR "cell saver" OR "intraoperative blood salvage" OR "autotransfusion" OR "red blood cell salvage" OR “RBC salvage” OR RBC reinfusion OR salvage transfusion OR salvaged RBCs OR “autologous transfusion”) |
| Embase  (n=1402) | (exp spinal fusion/ OR exp scoliosis/ OR exp kyphosis/ OR exp spinal curvature/ OR exp spine surgery/ OR spinal deformity.ti,ab,kw. OR adult spinal deformity.ti,ab,kw. OR spine surgery.ti,ab,kw. OR scoliosis.ti,ab,kw. OR kyphosis.ti,ab,kw. OR lordosis.ti,ab,kw. OR interbody fusion.ti,ab,kw. OR instrumented fusion.ti,ab,kw.)  AND  (exp erythrocyte transfusion/ OR exp autologous blood transfusion/ OR exp intraoperative blood salvage/ OR autologous blood transfusion.ti,ab,kw. OR cell salvage.ti,ab,kw. OR cell saver.ti,ab,kw. OR intraoperative blood salvage.ti,ab,kw. OR autotransfusion.ti,ab,kw. OR red blood cell salvage.ti,ab,kw. OR rbc salvage.ti,ab,kw. OR rbc reinfusion.ti,ab,kw. OR salvage transfusion.ti,ab,kw. OR salvaged rbcs.ti,ab,kw. OR autologous transfusion.ti,ab,kw.) |
| CENTRAL  (n=266) | ("spinal fusion" OR scoliosis OR kyphosis OR "spinal curvature" OR "spinal curvatures" OR "spine surgery" OR "spinal deformity" OR "adult spinal deformity" OR lordosis OR "interbody fusion" OR "instrumented fusion")  AND  ("erythrocyte transfusion" OR "blood transfusion" OR "autologous blood transfusion" OR "operative blood salvage" OR "cell salvage" OR "cell saver" OR "intraoperative blood salvage" OR autotransfusion OR "red blood cell salvage" OR "rbc salvage" OR "rbc reinfusion" OR "salvage transfusion" OR "salvaged rbcs" OR "autologous transfusion") |

**Supplementary Table 1*:* Search Strategies**

Full electronic search strategies used to identify relevant studies for this review, reported for each database. n = number of articles returned.

| **No.** | **Item** | **Outcome** |
| --- | --- | --- |
| 1 | Did the research questions and inclusion criteria for the review include the components of PICO? | Y |
| 2 | *Did the report of the review contain an explicit statement that the review methods were established prior to the conduct of the review and did the report justify any significant deviations from the protocol? | Y |
| 3 | Did the review authors explain their selection of the study designs for inclusion in the review? | Y |
| 4 | *Did the review authors use a comprehensive literature search strategy? | Y |
| 5 | Did the review authors perform study selection in duplicate? | Y |
| 6 | Did the review authors perform data extraction in duplicate? | Y |
| 7 | *Did the review authors provide a list of excluded studies and justify the  exclusions? | Y |
| 8 | Did the review authors describe the included studies in adequate detail? | Y |
| 9 | *Did the review authors use a satisfactory technique for assessing the  risk of bias (RoB) in individual studies that were included in the review? | Y |
| 10 | Did the review authors report on the sources of funding for the studies  included in the review? | Y |
| 11 | *If meta-analysis was performed did the review authors use appropriate  methods for statistical combination of results? | Y |
| 12 | If meta-analysis was performed, did the review authors assess the potential impact of RoB in individual studies on the results of the meta-analysis or other evidence synthesis? | Y |
| 13 | *Did the review authors account for RoB in individual studies when interpreting/ discussing the results of the review? | Y |
| 14 | Did the review authors provide a satisfactory explanation for, and discussion of, any heterogeneity observed in the results of the review? | Y |
| 15 | *If they performed quantitative synthesis did the review authors carry out an adequate investigation of publication bias (small study bias) and discuss its likely impact on the results of the review? | Y |
| 16 | Did the review authors report any potential sources of conflict of interest, including any funding they received for conducting the review? | Y |

**Supplementary Table 2: AMSTAR-2 quality appraisal**

AMSTAR-2 criteria applied to the present review, with item-level judgements (Y = yes; PY = partial yes; N = no). **= Critical weaknesses*

| **Outcome** | *k* | **SMD** | | | | **RoM** | | |
| --- | --- | --- | --- | --- | --- | --- | --- | --- |
|  |  | SMD (95% CI) | *P-v*alue | *τ²* | *I²* | RoM (95% CI) | *P-v*alue | *τ²* |
| Intraoperative volume | 15 | 0.86 (-0.24 to 1.96) | .11 | 3.40 | 99% | 0.74 (0.53 to 1.04) | .08 | 0.29 |
| Postoperative volume | 8 | 0.25 (0.04 to 0.46) | .026 | 0.00 | 0% | 0.67 (0.52 to 0.87) | .009 | 0.03 |
| Total perioperative volume | 9 | 1.15 (-0.75 to 3.05) | .20 | 5.58 | 99% | 0.71 (0.39 to 1.28) | .21 | 0.38 |

**Supplementary Table 3: Pooled transfusion volume estimates by effect measure**

RoM expressed as ICS relative to control (RoM <1 indicates lower transfused volume with ICS). Both measures pooled using a random-effects model, with RoM pooled on a log scale. CI = confidence interval; *k* = number of studies; RoM = ratio of means; SMD = standardized mean difference.

**Supplementary Table 4: Risk of bias assessment**

Summary of risk of bias assessment for non-randomised studies (ROBINS-I), reported by individual domain and overall risk of bias.

**Supplementary Table 5: Certainty of evidence (GRADE)**

GRADE assessment of certainty of evidence for each primary outcome in the present review.

| **Author** | **Critical Flaws** | **Non-Critical Flaws** | **Overall Confidence** |
| --- | --- | --- | --- |
| Liu et al. (2017) | **Item 2** - No explicit protocol registered before conducting the review  **Item 7** - No list of excluded studies provided  **Item 9** - No risk of bias assessment performed  **Item 13** - Risk of bias not considered in results  **Item 15** - Publication bias not considered | **Item 10** - Funding sources of included studies not reported  **Item 12** - Did not explore impact of RoB | **Critically Low** |
| Cheriyan et al. (2020) | **Item 2** - No explicit protocol registered before conducting the review  **Item 7** - No list of excluded studies provided  **Item 9** - Inappropriate risk of bias assessment performed - used Newcastle-Ottawa scale  **Item 13** - Risk of bias not considered in results | **Item 12** - Did not explore impact of RoB in discussion | **Critically Low** |
| Feuer et al. (2025) | **Item 2** - No explicit protocol registered before conducting the review  **Item 7** - No list of excluded studies provided  **Item 9** - No risk of bias assessment performed  **Item 13** - Risk of bias not considered in results | **Item 3** - No justification for included study designs  **Item 12** - Did not explore impact of RoB in discussion |  |
| Kale & Patel et al., 2025 (this review) | **None** | **None** | **High** |

**Supplementary Table 6: AMSTAR-2 quality appraisal of prior reviews**

Summary of AMSTAR-2 assessment of previously published systematic reviews on this topic, indicating critical and non-critical flaws, and overall confidence rating.

**A**

**B**

**C**

**Supplementary Figure 1: Patients requiring transfusion with versus without ICS use**

Forest plot comparing the risk ratio (random effects model) for the proportion of patients requiring transfusion during the (A) intraoperative, (B) postoperative, and (C) total perioperative periods between patients receiving (‘Salvage’) or not receiving (‘No Salvage’) intraoperative cell salvage. Individual study estimates are shown as squares proportional to study weight. Horizontal lines indicate 95% confidence interval. Diamond represents pooled estimate for overall effect. IV = inverse variance.

**Supplementary Figure 2: Total transfusion-related cost with versus without ICS use**

Forest plot comparing the mean difference (random effects model) in total transfusion-related costs for patients receiving (‘Salvage’) or not receiving (‘No Salvage’) intraoperative cell salvage. Individual study estimates are shown as squares proportional to study weight. Horizontal lines indicate 95% confidence interval. Diamond represents pooled estimate for overall effect. IV = inverse variance.

**Supplementary Figure 3: Hospital length of stay with versus without ICS use**

Forest plot comparing the mean difference (random effects model) in hospital length of stay (LOS) for patients receiving (‘Salvage’) or not receiving (‘No Salvage’) intraoperative cell salvage. Individual study estimates are shown as squares proportional to study weight. Horizontal lines indicate 95% confidence interval. Diamond represents pooled estimate for overall effect. IV = inverse variance.

**Supplementary Figure 4: Trial sequential analysis for primary outcomes**

Trial sequential analysis (TSA) for each primary outcome, showing the cumulative Z-score against the accrued cumulative sample size. (A) intraoperative transfusion volume; (B) postoperative transfusion volume; (C) total perioperative transfusion volume; (D) postoperative day 1 (POD1) hemoglobin; (E) POD1 hematocrit; (F) overall complications.

**(A) Intraoperative transfusion volume**

**(B) Postoperative transfusion volume**

**(C) Total perioperative transfusion volume**

**(D) Postoperative day 1 (POD1) hemoglobin**

**(E) Postoperative day 1 (POD1) hematocrit**

**(F) Overall complications**

**Supplementary Figure 5: Publication bias assessment**

Funnel plot of effect estimates from included studies plotted against their respective standard errors for the outcome of intraoperative transfusion volume requirement.
